# Improving transition readiness and quality of life (QOL) with a pediatric lupus health passport

**DOI:** 10.1186/1546-0096-10-S1-A23

**Published:** 2012-07-13

**Authors:** Emily Von Scheven, Lori B Tucker, Lakshmi N Moorthy, Erica F Lawson, Carolyn Neville, Deborah DaCosta, Paul R Fortin

**Affiliations:** 1 BC Childrens Hospital, Vancouver, BC, Canada; 2 Royal Victoria Hospital, Montreal, QC, Canada; 3 Toronto Western Hospital, Toronto, ON, Canada; 4 UMDNJ/RWJ Medical School, Metuchen, NJ, USA; 5 University of California San Francisco, San Francisco, CA, USA

## Purpose

Childhood onset Lupus (cSLE) is a chronic condition that usually requires life-long care, and optimizing self-management skills for youth with cSLE may improve health outcomes. This project aims to evaluate the impact of an educational intervention with a pediatric SLE-specific health passport (pLHP) for transitioning youth with cSLE.

## Methods

We adapted the pHLP from an adult LHP developed by the Canadian Network for Improved Outcomes in SLE (CaNIOS), by modifying the content and design to tailor to the needs of teens; and we then assessed acceptability through pilot testing and structured interviews. Patients with cSLE age 14-21 years recruited from three North American centers were instructed on the use of the pLHP. Transition readiness was assessed by a modified version of the California Healthy and Ready to Work Transition Health Care Guide, self-efficacy by a modified version of the Children’s Arthritis Self-Efficacy Scale, medication adherence by the MASRI, and SLE knowledge by a tool developed by the investigators. Baseline data is presented here.

## Results

48 patients aged 14.4 -21.2 yrs (mean 17.8 yrs), 79% female, with mean age of diagnosis of 13 yrs (range 7 -17 yrs) and mean disease duration of 5 years (range 0.4-12 yrs) were evaluated. Current medications included plaquenil (85%), mycophenolate (59%), rituximab (11%), azathioprine (7%), methotrexate (7%), anti-depressant (14%) and history of cyclophosphamide (29%). Median disease activity at enrollment by SLEDAI was 0 (range 0-14); median cumulative damage by SLICC/Damage Index was 0 (range 0-2). On an SLE knowledge test of 12 true/false questions the median score was 80% correct (range 5-100%) and on a 10 question fill-in-the-blank knowledge test, the median score was 60% (range 10-90%). Age was associated with higher knowledge on the true/false test (r=0.6, p=0.0025). Self-efficacy scores varied widely (mean 54, range 38-65). Transition readiness (assessed with a 3-point likert scale; 1= I am doing this, 2=I need some help, 3=someone else does this for me) revealed a mean score across all tasks of 1.5 suggesting that many patients were dependent on others to complete health care tasks. Overall transition readiness was associated with increased age (r=0.6, p=0.001). Self-reported adherence for taking prednisone was good: 35% indicated taking all medication doses, 23% indicated taking 90% of doses, 10 % indicated taking 85%, and none reported less than 60% adherence. To date, 6 patients have undergone a 6 month follow-up visit and our preliminary impression is that although most patients agree that the pLHP is informative, many are not routinely entering their personal disease-related information.

## Conclusion

Teens with SLE have variable self-efficacy and are in need of education about their disease and their medications. Many teens with cSLE still rely on their parents for assistance with medical tasks. We are continuing our follow-up visits and anticipate that the pLHP will significantly bridge gaps in knowledge and self-efficacy and positively impact future self care and health outcomes.

## Disclosure

Emily Von Scheven: None; Lori B. Tucker: None; Lakshmi N. Moorthy: None; Erica F. Lawson: None; Carolyn Neville: None; Deborah DaCosta: None; Paul R. Fortin: None.

